# Exercise Interventions to Address Sarcopenia in People with Multiple Myeloma: A Scoping Review

**DOI:** 10.3390/curroncol32100581

**Published:** 2025-10-18

**Authors:** Leslie P. Ternes, Graeme M. Purdy, Stéphanie Bernard, Margaret L. McNeely

**Affiliations:** 1 Department of Physical Therapy, Faculty of Rehabilitation Medicine, University of Alberta, Edmonton, AB T6G 2G4, Canada; lternes@ualberta.ca (L.P.T.); gmpurdy@ualberta.ca (G.M.P.); stephanie.bernard@fmed.ulaval.ca (S.B.); 2 École des Sciences de la Réadaptation, Université Laval, Québec, QC G1V 0A6, Canada; 3 Supportive Care and Patient Experience, Cancer Care Alberta, Edmonton, AB T6G 1Z2, Canada

**Keywords:** sarcopenia, exercise, cancer, muscular strength, multiple myeloma, scoping review

## Abstract

**Simple Summary:**

Sarcopenia, a condition involving loss of muscle strength and quality, is common in people with multiple myeloma. Exercise may help counteract these effects, but research in this area is limited. We reviewed published studies to see how exercise has been studied for people with multiple myeloma and whether sarcopenia outcomes were included. Twelve studies met our criteria, involving 967 participants (624 with multiple myeloma). While all studies measured physical performance, fewer measured muscle strength, quantity, or quality, and none directly assessed sarcopenia as defined by international standards. Some studies found that exercise improved performance, muscle strength, and quantity, but the results varied widely. Differences in exercise programs, outcome measures, and the timing of interventions made comparisons difficult. More large, well-designed studies are needed to understand the role of exercise in preventing or treating sarcopenia in people with multiple myeloma.

**Abstract:**

**Background**: The clinical characteristics of sarcopenia, including low muscular strength, are commonly seen among people with multiple myeloma. A scoping review was conducted to explore the role of exercise as a potential countermeasure for sarcopenia in this population. Our objectives were to (1) describe the design and findings of the studies and (2) identify the outcomes used in exercise-related studies to characterize sarcopenia. **Methods**: A systematic search (to March 2025) was conducted for published studies involving exercise or physical activity for individuals with multiple myeloma using key databases (MEDLINE, Embase, CINAHL, Scopus). **Results**: Of 971 articles reviewed, 12 articles were included, involving 967 total participants and 624 with multiple myeloma. All 12 studies included a measure for muscle physical performance, 9 studies included measures for muscular strength, and 7 studies included measures for muscle quantity/quality. Five studies reported a significant improvement from exercise for measures of muscular strength, four studies reported a significant benefit for physical performance, and three studies reported a benefit in muscle quantity. Few studies included outcomes that met all the international criteria recommended to characterize sarcopenia. **Conclusions**: Further multicentre research trials are needed to better understand whether and how exercise may be helpful for people with multiple myeloma, especially in the context of sarcopenia.

## 1. Introduction

Hematological malignancies such as leukemia, lymphoma, and multiple myeloma (MM) represent roughly 7–9% of all cancers and account for over 1.2 million new cancer diagnoses yearly in economically developed regions of the world [1,2]. MM is a cancer of the plasma cells, and although incurable, advances in treatment over the last 20 years have significantly improved the length of life of people living with MM [3]. Despite these improvements, older adults with MM experience poorer treatment outcomes than their younger counterparts due to the increased prevalence of comorbidities and frailty seen with advancing age [4,5].

Sarcopenia is characterized by a progressive loss in the quantity and quality of muscle fibers, leading to reduced muscle mass and strength [6]. The pooled prevalence of sarcopenia in elderly populations ranges from 10% to 40% [7]. While age-related declines in muscle strength and quality are expected, these losses are accelerated in older adults with cancer compared to those without cancer [8]. Although most commonly associated with aging, sarcopenia also affects sedentary individuals and people with comorbidities that compromise musculoskeletal function or restrict physical activity (e.g., arthritis) [9]. Among individuals with MM, the presence of sarcopenia is associated with poorer physical functioning and quality of life and an increased risk of falls and fractures [6,10]. Moreover, loss of muscle mass is a predictor of an increased risk of chemotherapy toxicity and poorer overall survival [11]. Hematopoietic stem cell transplantation (SCT) is a common treatment for MM and is associated with better long-term survival [12]. For those undergoing autologous SCT, sarcopenia is highly prevalent, with one study reporting a rate of 51% [5]. While the burden of sarcopenia is particularly high among elderly individuals with MM, the influence of disease- and treatment-related factors across all age groups highlights the importance of examining sarcopenia in the broader MM population.

Exercise interventions have been extensively studied in patients with solid tumors, such as breast, prostate, and colorectal cancers, as well as in hematological cancers of leukemia and lymphoma [13]. In these populations, supervised and resistance-based exercise programs consistently demonstrate improvements in muscle strength, aerobic capacity, fatigue, and overall quality of life [13]. Lifestyle interventions including exercise are also the cornerstone treatment for sarcopenia [14,15,16]. Exercise, particularly resistance training, helps maintain physical function during normal aging and contributes to the preservation of lean mass during and after cancer treatment [17,18,19]. In individuals with MM, concerns over safety predominate, especially given their proportionately high risk of fracture, and may be seen as a barrier to exercise [20]. Due to these concerns over safety, there is a paucity of exercise-related research in this cancer population [20]. In a recent systematic review led by Nicol and colleagues (2023), exercise interventions were found to be safe and well attended by individuals with MM, with some isolated studies showing a benefit for muscular strength, peak oxygen consumption, and physical activity [21].

At present, there is no clear definition, nor clear clinical cut-offs, for the diagnosis of sarcopenia, which poses difficulties in assessing the impact of exercise on sarcopenia. The European Working Group on Sarcopenia in Older People 2019 (EWGSOP2) defines sarcopenia as a progressive and generalized skeletal muscle disorder characterized by low muscle strength (probable sarcopenia), confirmed when low muscle quantity or quality are also present, and considered severe when poor physical performance is additionally observed [22]. Given that sarcopenia appears to disproportionately affect those with MM [5], developing targeted interventions to address muscle loss may be integral in addressing the quality-of-life issues of these individuals. However, sarcopenia is not routinely identified or diagnosed in clinical MM practice, and when it is studied, heterogeneous definitions (mass, strength, or performance) make it challenging to compare findings across studies [23]. Therefore, this scoping review aimed to explore the current literature concerning exercise interventions addressing sarcopenia in individuals with MM and to identify gaps in the research. In addition, we sought to categorize the measures and measurement methods used to investigate sarcopenia and assess their alignment with EWGSOP2 recommendations. The findings will help to inform the planning and design of upcoming research focusing on investigating the effects of exercise interventions on sarcopenia in individuals diagnosed with MM.

## 2. Materials and Methods

### 2.1. Design

For this study, a scoping review methodology was selected to explore the literature due to the relative infancy of sarcopenia-related studies in the MM literature [24,25] and to identify gaps and future directions. This scoping review follows the formal guidance of the Joanna Briggs Institute (JBI) method and was registered with Figshare [26]. The reporting in this article follows the Preferred Reporting Items for Systematic Reviews and Meta-Analyses extension for scoping reviews (PRISMA-ScR) statement [27]. The protocol for this scoping review was registered with Figshare and is available at https://figshare.com/articles/journal_contribution/Multiple_Myeloma_and_Sarcopenia_Scoping_Review_Protocol/21989534?file=39445759 (accessed on 1 July 2025).

### 2.2. Selection Criteria

The research team included a graduate student with expertise in exercise physiology (L.T.), a medical student with a background in kinesiology (G.P.), and two oncology rehabilitation clinical researchers experienced in systematic and scoping review methodology (S.B., M.L.M.). The team met to outline the study objectives and inclusion criteria before conducting the systematic search. Inclusion criteria for this review are described based on the type of participants (population), concept (outcomes and intervention), and context framework [25]. Articles were included if they met the following inclusion criteria:

1. Population:

Adults (18 years of age or older) with a cancer diagnosis of MM at any stage of disease and at any point in the cancer treatment trajectory defined from the point of diagnosis throughout the treatment journey were included. This criterion included individuals who were currently undergoing treatment, had undergone an SCT, or were post-treatment. Studies were included if the population sample included any people diagnosed with MM, given that individuals with MM are generally an under-represented group in the literature.

2. Interventions:

Studies that employed an exercise intervention, or a physical activity goal, were included. The definition of exercise used for this study was any planned, structured, repetitive, and purposive bodily movement produced by the skeletal muscles that results in increased energy expenditure, intending to improve or maintain physical fitness [28]. The types of exercise eligible for the review included cardiovascular (aerobic) exercise regimens and strength/resistance exercise regimens, as well as interventions to increase step counts or physical activity minutes. The exercise program could be supervised or unsupervised and delivered to participants in group classes or as individual prescriptions. Eligible locations included inpatient or outpatient hospital settings, clinic- or community-based sites, or the individual’s home. There were no limits on other program characteristics such as frequency, intensity, type, time, or program duration.

3. The outcomes aligned with the conceptual definition of sarcopenia:
  For this review, the definition of the European Working Group on Sarcopenia in Older People 2019 (EWGSOP2) was adopted. Studies were required to include a measure aligning with one or more of the following outcomes:
  - Muscle strength: Grip strength for measurements of muscular strength of the upper body or either the chair stand test for lower body muscular strength (5 repetitions for time) or the timed chair stand test (as many repetitions in 30 s) for the lower body [22].
  - Muscle quantity and quality: Either direct imaging through magnetic resonance imaging (MRI) or computed tomography (CT) or body composition measurements of interest, including dual-energy X-ray absorptiometry (DXA), bioelectrical impedance analysis, body mass index, and calf circumference [22].
  - Physical performance: Gait speed, short physical performance battery (SPPB), and the timed up and go (TUG) test [22].
  - Alternative tests: For this scoping review, any alternative measurement types aligning with these outcomes were considered if deemed relevant by the review authors.

4. Study designs: All study designs were included except for studies exclusively focused on a qualitative methodology or case studies.
5. Language restrictions: Only English- or French-language publications were considered.
6. Publication types: Only peer-reviewed, full-length articles were included. Grey literature, including conference abstracts and other non-peer-reviewed articles, were excluded.
7. Context: No limitations were placed on geographical or locational factors or factors related to culture, race, ethnicity, sex, or gender.

### 2.3. Search Strategy

A health sciences librarian, in conjunction with the research team, developed the search strategies for the four electronic databases (Medline, CINAHL, Embase, and Scopus). An initial search using key terms (“multiple myeloma”, “sarcopenia”, “exercise”) along with associated terms and synonyms was conducted using Medline to analyze free text words contained in relevant titles and abstracts, as well as to identify controlled vocabulary used to describe the articles. This initial search informed the final search strategy. The databases were searched from the date of inception to 31 March 2025. Details on the search strategy for each database is available in Supplementary Material S1.

### 2.4. Study Selection

After applying the search strategy, all retrieved articles were uploaded into Covidence Systematic Review Software 2025 (Veritas Health Innovation, Melbourne, Australia), and duplicates were removed. The titles and abstracts of retrieved articles were independently reviewed by two members of the research team (L.T., G.P., or S.B.). Reviewers met regularly to discuss progress and resolve any discrepancies through the consensus of a third reviewer (M.L.M.). Upon the completion of title and abstract screening, the remaining articles were advanced to full-text screening. Full texts were independently reviewed by two reviewers for inclusion (L.T., G.P., S.B., or M.M.). Researchers also hand-searched the reference lists of included articles. The same process of resolving conflicts during the title and abstract screening stage was applied. Full-text articles that met the inclusion criteria were included in the review, and the details from these articles were extracted. Inter-rater reliability was assessed using Cohen’s kappa [29].

### 2.5. Data Extraction

Data were abstracted from the full texts of the included studies. A standardized form was used to collect data on the study characteristics (author, country of publication, year of publication, study design), study population characteristics (sample size, medical characteristics, sex and age of participants), and intervention characteristics (frequency, intensity, type of exercise, supervision level, adherence, study completion). Exercise regimens were coded as (1) supervised, (2) partially supervised if there was a combination of self-directed home exercise sessions and sessions guided by an exercise professional, or (3) self-directed for regimens with no or minimal supervision (less than 15% of sessions).

Data abstracted for outcomes relevant to characterizing key indicators associated with sarcopenia were evaluated as per the EWGSOP2 criteria [22]. For the purposes of this review, alternative testing methods were accepted if the review authors considered the test equivalent to meeting the criteria of the EWGSOP2 in terms of measuring physical performance, muscular strength, or muscle quality or quantity.

## 3. Results

### 3.1. Search Results and Study Characteristics

**Figure 1:**
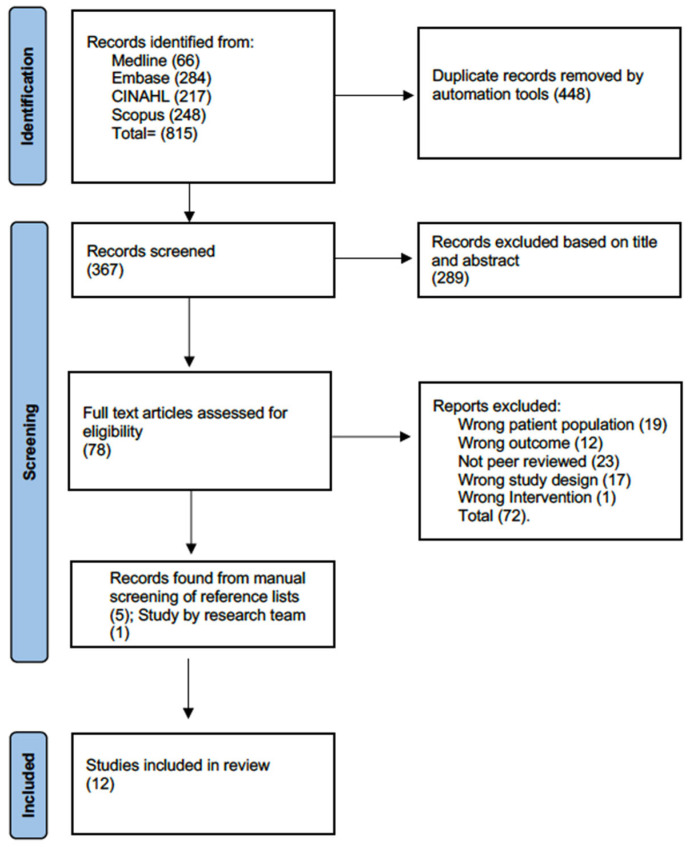
Flow diagram.

The search resulted in a total of 971 articles (see Figure 1: Study Flow). After the removal of duplicates, 421 articles were screened based on the title and abstract, from which 80 full-text articles were reviewed. Of these, six articles met all inclusion criteria and were included [30,31,32,33,34,35]. Six additional articles were identified, with five found through hand-searching of the reference lists of the included articles [36,37,38,39,40] and one involving a recent publication led by our team [41]. A total of 12 articles were included, with 967 participants. Cohen’s kappa for the agreement between reviewers was 0.76 (substantial agreement).

The included studies were diverse in design, including five randomized controlled trials [31,34,36,38,40], two retrospective cohort studies [30,33], four single-group before-and-after studies [32,35,37,41], and one study using a modified Zelen design with an embedded randomized control trial [39]. Of the 967 participants, 624 (64.5%) had a diagnosis of MM. The sample sizes ranged from 24 to 205 participants, with the number of participants with MM ranging from 1 to 187 participants.

### 3.2. Participant Characteristics and Settings

Details on the study design, participant characteristics, and intervention location are provided in Table 1. Seven studies included participants with a variety of cancers [30,32,33,35,36,38,40], while five included only individuals with MM [31,34,37,39,41]. All studies included both male and female participants, and where reported, most studies were found to include a higher proportion of male participants [30,31,32,34,37,38,39,40]. Overall, the age of the participants ranged from 20 to 83 years. Ethnicity was not consistently reported across all studies; however, where reported, studies largely comprised white participants [31,33,34,38,39,41].

Among the included studies, the exercise intervention was conducted at varying times in the treatment trajectory. Three studies involved the period encompassing both pre-SCT and post-SCT [30,31,33]. Two studies were conducted with participants who were pre-SCT receiving chemotherapy [32,34], and two studies included participants who were post-SCT [38,40]. Two studies involved participants who were off treatment or on maintenance therapy, irrespective of receiving an SCT or not [37,39]. One study included participants who were SCT ineligible, post-SCT, or had relapsed [41]. One study included participants who had completed chemotherapy within 4 weeks, with some receiving radiation therapy, and some who were post-SCT [36]. One study involved participants at any time point if they were actively receiving cancer treatment [35]. The studies employed diverse exercise delivery models: exclusively home exercise programming [31,34,41]; gym exercise programming with or without a home-based option [36,37]; inpatient hospital sessions [32,33]; physiotherapy-clinic-based programming [40]; a combination of home-based exercise and inpatient hospital sessions [30,39]; and a combination of home-based and clinic-based sessions [35,38].

Several studies reported statistically significant improvements in muscle strength, physical function, or performance outcomes. Furzer et al. demonstrated increases in leg press and chest press strength following a 12-week, partially supervised exercise program delivered during active treatment [36]. Similarly, Groeneveldt et al. and Koutoukidis et al. reported significant gains in muscular strength and functional performance among MM survivors in the maintenance/recovery phase, both using multimodal exercise programs with resistance training components [37,39]. Rosko et al. found improvements in gait speed and SPPB scores in patients undergoing active therapy following a supervised and home-based hybrid intervention [35]. Finally, Purdy et al. observed functional gains in sit-to-stand and step testing in a sample of transplant and non-transplant MM patients completing a partially supervised, individualized exercise program [41]. Across these studies, common features included the integration of resistance training, the delivery of exercise in either supervised or hybrid formats, and implementation during or following periods of active treatment.

### 3.3. Exercise Interventions

Overall, five studies were partially supervised [35,37,38,39,41], four articles had unsupervised exercise sessions [30,31,34,36], and three studies had fully supervised exercise sessions [32,33,40] (Table 2). The frequency of exercise per week varied across studies. Four studies involved a frequency of three times per week [36,37,38,39], four studies prescribed daily exercise [30,31,32,34], three studies were conducted twice a week [35,40,41], and one study was conducted five times per week [33].

The exercise interventions varied greatly across all studies, with ten studies involving both resistance and aerobic exercise programs [30,31,33,34,35,36,37,39,40,41], one study involving resistance exercise only [38], and one study involving aerobic exercise only [32]. Five articles included stretching into their regimen either as a warm-up or a cool-down [31,34,35,37,41], and two included balance training [35,41].

For the studies involving resistance exercise, various methods and levels of intensity were prescribed. Two studies used the Borg 10-point rating of perceived exertion (RPE) scale, specifying a goal of 3 to 5 (i.e., light to somewhat hard) [30,41], and two studies used the Borg 20-point RPE scale, with one specifying a goal of 13 (i.e., somewhat hard/termed moderate exercise) [38] and the other specifying an RPE of 15–17 (i.e., hard to very hard) [34]. Three studies used the percentage of 1RMs for resistance exercise with varying goals of intensities; 50% 1RM [36], 65–80% 1RM [40], and 60–80% 1RM [34]. Three studies prescribed intensity based on other methods, such as using colour-coded bands for varying levels of resistance [31], progression from low weights on machines or using light bands and slowly progressing over time [37], and using a prescription based on the participant’s 10-repetition maximum [39].

For the studies involving aerobic exercise, the prescription varied considerably among the included studies. Percentage of heart rate maximum (HRmax) was the most common way to prescribe intensity for aerobic exercise; one study used 70% HRmax [32], one study used 65% HRmax [40], one study used 50% HRmax [36], one study used a range of 65–80% HRmax [34], and one study used a range of 50–75% HRmax [39]. One study prescribed intensity based on the heart rate reserve (HRR), set at 50% of the HRR [37]. Three studies used the Borg 20-point RPE scale, with one study prescribing an RPE of 10–13 (very light to somewhat hard) [36], one study an RPE 12–15 (light to hard) [31], and another study an RPE 11–13 (fairly light to somewhat hard) [34]. One study used both the “talk test” and an RPE of 3 to 5 (easy to somewhat difficult) on the Borg 10-point RPE scale [41]. Two studies used the same intensity prescription for their aerobic and resistance training, with one using the term “moderate” intensity [35], and the other using metabolic equivalents (METs) set at 3–4 METs (i.e., light- to moderate-intensity) [33].

### 3.4. Outcome Measures

#### 3.4.1. Muscle Strength

Eight studies [31,32,33,35,37,38,39,40] included measures of muscle strength for the upper and lower body, and one study included a measure of lower body strength alone [41]. Handgrip dynamometry was the most common test used to assess muscular strength in the upper body [33,35,37,38,39,40], and the chair sit-to-stand test for the lower body [35,36,38,40,41]. Five studies conducted repetition maximum testing for lower body muscular strength, with two studies using the 10-repetition maximum [37,39], two studies using a 1-repetition maximum test [31,33], and one study using a 5-repetition maximum test [36]. Two studies performed multi-joint testing for both the upper and lower body measures [31,36]. One study included a measure of multi-repetition arm curls in 30 s [38]. In terms of the findings, five studies reported statistically significant increases in muscular strength [35,36,37,39,41], and one study reported a statistically significant decrease in muscular strength [33].

#### 3.4.2. Muscle Quality

Seven studies examined muscle quality through measures of body composition; two studies used bioelectrical impedance [37,39], one used DXA scanning [36], one used thigh ultrasound [38], one study used skinfolds [40], one used circumference measurements [33], and one used the Bod Pod [31]. Three studies reported statistically significant changes in muscle quality, with two studies reporting significant improvements [31,36] and one reporting a decrease in muscle quality [33].

#### 3.4.3. Muscle Performance

All 12 studies included in this review included one or more measures of physical performance. Aerobic capacity was the most performed measure, with two studies completing a test of the peak oxygen uptake (VO_2peak_) on a cycle ergometer [39,40], two completing submaximal treadmill tests [31,32], one completing a submaximal cycle ergometer test [36], and three involving submaximal walking tests with one eight-minute [37] and two six-minute walk tests [33,34]. Three studies implemented the timed up and go [30,36,38], two involved the stair climb test [30,36,38], and one study included a 15-foot walk time test [30,38]. Three studies reported statistically significant improvements in muscle performance [30,35,41].

### 3.5. Alignment with EWGSOP2 Criteria

#### 3.5.1. Definition of Sarcopenia or Related Terms

Table 3 provides details on articles that provided a definition of sarcopenia or similar terms, as well as whether the study outcomes met the criteria of the EWGSOP2. One study [33] provided criteria for an individual to be considered sarcopenic, while another mentioned sarcopenia but did not include a definition [38]. The other articles, while they did not mention sarcopenia, used similar terms, including frail [27,30,35], bone frailty [37], muscle wasting [31,34], loss of muscle mass and cachexia [32], and physical deconditioning [30,38].

#### 3.5.2. Measurements for Sarcopenia

Nine studies included appropriate tests for muscular strength as per the criteria of the EWGSOP2 (Table 3) [31,33,35,36,37,38,39,40,41]. Six articles included appropriate tests for muscle quantity or quality [31,33,36,37,38,40], with one using a proxy measure (calf circumference) [33]. Three studies met the criteria for muscle performance [30,35,38], while the remainder included tests deemed to be reasonable alternatives (coded as partially meeting the criteria for testing muscle performance) [31,32,33,34,36,37,39,40,41]. One study used skinfolds as a proxy for measurement of percent body fat [40]. Four studies measured aerobic capacity through maximal [39,40] and submaximal treadmill or cycle ergometer tests [31,32].

#### 3.5.3. Adverse Events

Adverse event reporting varied across the included studies (Table 2). All events were resolved without hospitalization, and none met the criteria for a serious adverse event.

## 4. Discussion

One of the main findings of this scoping review was the heterogeneity across studies in terms of the timing of the intervention, chosen exercise parameters, and types of outcome measures—factors that preclude us from drawing clear conclusions on the state of the evidence. Similar to the findings of a recent systematic review by Goodhew et al. (2023), the benefits seen from exercise in individuals with MM differed across studies and outcomes [20]. Seven of the studies included in this review demonstrated a positive response to exercise in one or more of the categories of muscular strength, muscle quantity and quality, and muscle performance. Even though the optimal exercise parameters are still unclear, these findings support the rationale that exercise may be a potential tool in terms of rehabilitation or prehabilitation. Moreover, while not all studies demonstrated a clinically significant improvement with exercise or a positive response across all outcomes, this may not necessarily indicate a lack of benefit. In the context of the MM population, with highly toxic treatment regimens, an attenuation in the loss of muscle mass, for example, may be considered a positive outcome. Of note, only two studies with eligibility restricted to MM had a sample size > 100, highlighting the need for large-scale multicentre trials in this population [34,39].

Of all included studies in this review, 25% implemented completely supervised exercise programs. There are several advantages of supervised exercise for people with MM, including closer monitoring for safety, exercise attendance, and adherence to exercise prescription variables [42]. A recent systematic review noted the lack of reporting of exercise adherence in older participants with sarcopenia, with only 20% of articles reporting the actual adherence to the prescription variables of frequency, intensity, type and time (FITT variables) [43]. The adherence rates for group exercises for fall prevention in older adults were 74% for supervised programming as compared to 21% for home-based exercises, demonstrating advantages when receiving supervision or guidance [43,44]. Moreover, self-reporting of adherence for home-based unsupervised programming is susceptible to memory recall and social approval or other biases [43,44]. Researchers should consider following the existing guidelines and checklists (e.g., TIDieR Checklist) when reporting their exercise interventions in further trials [45].

For individuals with MM, the importance of supervision also extends to safety considerations. In particular, the presence, location, and extent of bone lesions or recent fractures are critical to determining safe exercise prescription, yet these details were rarely reported in the included studies. While the exercise interventions appeared to be safe and generally well tolerated, the reporting of adverse events was also limited. Where reported, adverse events were mostly musculoskeletal in nature and resolved without lasting effects. However, the lack of standardized adverse event monitoring and reporting limits our ability to fully evaluate exercise-related risks in the MM population. Future trials should prioritize adverse event reporting to ensure that both the efficacy and safety of exercise interventions are clearly documented [46]. Supervised programming, whether delivered in person or virtually, may address these limitations and help mitigate risk while supporting exercise adherence.

Resistance training is recognized as a potential countermeasure for sarcopenia [47,48] and was a common component among the studies in this scoping review. As MM is a cancer that is diagnosed largely in older adults [49], concerns over sarcopenia are particularly relevant. Sarcopenia is prevalent among older, apparently healthy populations after the age of 50 years, with gradual deteriorations of 1–2% and 2.5–4% per year in muscle mass and strength, respectively [50]. Sarcopenia in older adults, including those with MM, is associated with an increased risk of falls, fractures, and hospitalization and higher mortality rates [43]. While the optimal exercise prescription has not yet been defined in the MM population, the findings of a recent scoping review of older adults in the general population suggest a benefit from resistance training programs [48]. In their review of studies involving older adults, the most common exercise prescribed for sarcopenia encompassed a total of eight exercises for the large muscle groups of both the lower and upper body and with resistance applied using weight plates, resistance machines, or barbells [48]. The most common prescribed intensity started at 50% of 1RM, with a gradual increase to 80% of 1RM and a frequency of three times a week, for a duration of 12 weeks. The authors reported favorable findings for muscular strength and body composition, with strength improvements seen in handgrip strength, timed up-and-go, chair stand time, stair climb, and overall 1RM and body composition improvements for waist–hip ratio, lean body mass, and fat mass [48]. While the studies in our review varied considerably in the prescription variables, four of the five studies conducted specifically with the MM population reported significant improvements in outcomes related to muscle strength, quantity and quality, and performance, suggesting that exercise of any type or intensity may be protective against sarcopenia.

In studies that included mixed hematological malignancy populations, the outcomes were generally reported for the entire cohort, with limited reporting of the results for the MM subgroup. As a result, it was not always possible to determine whether the observed effects applied to the MM participants. This limitation highlights the need for future trials designed exclusively for MM populations or with sufficient subgroup analyses to enable MM-specific interpretation. When stratified by intervention type, supervised or hybrid programs [36,37,39,41] more consistently demonstrated significant improvements, whereas fully unsupervised programs [30,34] reported few or no meaningful changes. These findings suggests that the presence of supervision, even in partial or hybrid formats, may be critical to achieving positive outcomes in the MM population.

We found that few of the retrieved studies included a comprehensive evaluation using objective indicators of sarcopenia that met the standards of the EWGSOP2. Research on the effects of exercise on sarcopenia in people with MM is currently limited by the validity and robustness of the measures used to characterize the condition. For this reason, there is uncertainty about the effects of exercise on sarcopenia among individuals with MM. For example, skinfold measurements provide data related to subcutaneous fat and can serve as a marker to provide information on overall body composition but are not a direct measure of muscle quality [51]. While the EWGSOP2 includes many options within the categories of muscular strength, muscular quantity and quality, and muscle performance, gold-standard outcomes are preferred in the research setting—outcomes of value given their improved accuracy and reliability [52]. Tools considered to be gold standards for measuring muscle quality and quantity are magnetic resonance imaging (MRI), computed tomography (CT), and a four-compartment model of body composition [53]. No studies retrieved in this review included a gold-standard test to evaluate muscle quality and quantity—a key consideration for future research to understand the effect of exercise on sarcopenia in this population better.

### 4.1. Limitations and Strengths

We acknowledge that this scoping review is not without limitations. First, only studies published and written in either English or French were included. Second, five articles were found during our manual screening of the reference lists of included articles, suggesting our search strategy may have been too narrow given our focus on terms related to sarcopenia. While it is possible that further studies not explicitly focused on muscle mass or sarcopenia were missed by our initial search, such studies would also likely have lacked the detailed outcomes relevant to this review. As sarcopenia was not a primary focus of many of the included articles, the findings related to sarcopenia were also limited. Thus, to capture studies relevant to MM and exercise, broader searches are needed. In addition, one included study was conducted by two of the authors of this scoping review (G.P., M.M.); however, screening for inclusion and the data abstraction were verified by the two other authors (L.T., S.B.). Strengths of this review include the comprehensive and standardized review methods, the experience of the research team, and the unique focus on sarcopenia.

### 4.2. Summary and Future Directions

**Figure 2:**
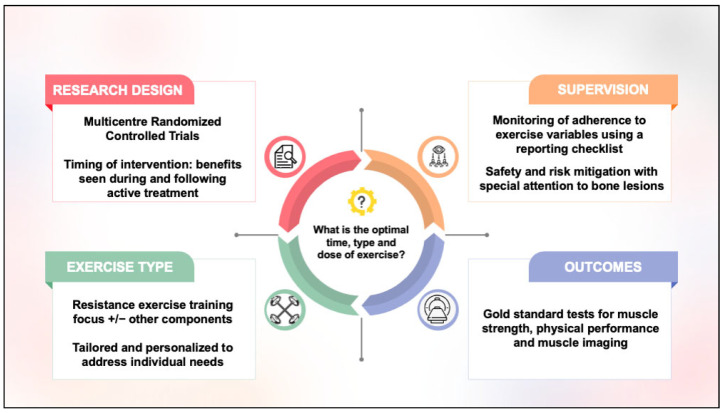
Considerations for future research.

To improve the quality of research in this field, we propose four considerations for future directions (Figure 2): (1) Given the low prevalence of MM compared to other common cancers, multicentre randomized controlled trial designs should be considered to support timely recruitment and evaluate the optimal timing of exercise across the treatment trajectory. (2) To understand response to exercise better, supervised exercise programs are needed, with closer attention paid to the monitoring and reporting of adverse events, exercise attendance, and exercise FITT prescription variables. (3) Tailored and personalized resistance exercise training programs, aligning with the evidence from older adult populations, are recommended to specifically address sarcopenia in MM. (4) A consensus on a core outcome set with consideration given to use of gold-standard imaging tests for muscle quality and quantity is needed to evaluate exercise outcomes best. Research that considers these identified gaps will help to progress our understanding of how exercise may benefit sarcopenia in people with MM. As more evidence becomes available, the next step will be to translate these findings into standard practice across the treatment trajectory. This could include establishing clear referral pathways for clinicians, tailoring exercise programs to patients with bone lesions or recent fractures, prioritizing supervised interventions where appropriate, and incorporating sarcopenia measures as practical screening tools into both clinical and community health settings.

## Figures and Tables

**Table 1:** Data extraction sheet of included studies: study design and population characteristics.

Author, Country, Year	Study Design	Sample Size/ No. with MM	Sex (M:F)	Age (Years)	Treatment Pathway	Intervention Location
Andres et al. USA 2020 [30]	Retrospective Groups based on KPS	*N* = 205 MM *n* = 87 (42%)	High-performance group (61:30), low-performance group (69:45)	High-performance group: 57.1 ± 14.1; low-performance group: 60.5 ± 10.6	Transplant (67 allogeneic, 138 autologous); pre- to 60 days post-SCT	Home-based program pre- and post-SCT; inpatient hospital during SCT
Dimeo et al. Germany 2003 [32]	Single Group	*N* = 66 MM *n* = 8 (12%)	34:32	45 ± 15 (20–73)	Induction + transplant (conventional chemo *n* = 45; high-dose chemo with SCT *n* = 21)	Inpatient hospital
Furzer at al. Australia 2016 [36]	Randomized Controlled Trial	*N* = 37 MM *n* = 4 (11%)	Not reported	48.9 ± 13.1 (22–68)	Transplant/active treatment (within 4 weeks of treatment)	Self-directed, gym-based exercise with physiologist support
Hacker et al. USA 2016 [38]	Randomized Controlled Trial	*N* = 67 MM *n* = 28 (42%)	41:26	53.3 ± 12.2	Transplant (autologous *n* = 39, allogeneic *n* = 26, haplo-identical *n* = 2); post discharge	Home-based and outpatient clinic
Persoon et al. Netherlands 2017 [40]	Randomized Controlled Trial	*N* = 109 MM *n*= 58 (53%)	69:40	EX = 53.5 (20–67) UC = 56 (19–67)	Transplant Recovery (autologous SCT; 6–14 weeks post-SCT if recovered)	Physiotherapy clinic
Rosko et al. USA 2021 [35]	Single Group	*N* = 30 MM *n*= 19 (63%)	13:17	62–83	Active Treatment	Physiotherapy clinic and home-based
Tanaka et al. Japan 2017 [33]	Retrospective	*N* = 34 MM *n* = 1 (2.9%)	Not reported	Mean: 47.4 (19– 67)	Transplant (allogeneic SCT conventional or reduced intensity)	Inpatient hospital
Coleman et al. USA 2003 [31]	Pilot Randomized Controlled Trial	*N* = 24	14:10	Male = 48–74 (Mean = 58.43) Female = 42–66 (Mean = 52.3)	Transplant (high-dose chemo + autologous SCT; 3 months pre- to 3 months post-SCT)	Home-based
Coleman et al. USA 2012 [34]	Randomized Controlled Trial	*N* = 187	UC: 55:37; EE: 54:41	UC = 56.4 ± 9.3 EX = 56.0 ± 10.5	Induction + Transplant (Total Therapy II. ^A^ Total Therapy III. ^B^)	Home-based
Groeneveldt et al. United Kingdom 2013 [37]	Single Group	*N* = 49	26:19	Median: 61 (46–74)	Maintenance (Post-chemo, off treatment or maintenance)	Outpatient gym- and home-based
Koutoukidis et al. United Kingdom 2020 [39]	Modified Zelen Design: Embedded Randomized Control Trial	*N* = 131	72:59	EX = 64 (35–86) UC = 63 (40–80)	Maintenance	Months 1–3: hospital- and home-based; months 4–6: home-based
Purdy et al. Canada 2022 [41]	Single Group	*N* = 28	14:14	65.0 ± 8.4	Mixed pathways (transplant-ineligible, first-line; post-SCT; or recurrent)	Virtual home-based

MM: multiple myeloma; SCT: stem cell transplant; EX: exercise group; UC: usual care group; KPS: Karnofsky Performance Status. ^A^ Total Therapy II included induction chemotherapy with VAD (vincristine, doxorubicin, and dexamethasone), DCEP (dexamethasone, cyclophosphamide, etoposide, and cisplatin), and CAD (cyclophosphamide, doxorubicin, and dexamethasone) and stem cell collection before high-dose melphalan, followed by a PBSC transplantation. ^B^ Total Therapy III included two cycles of VDTPACE (bortezomib, dexamethasone, thalidomide, cisplatin, doxorubicin, cyclophosphamide, and etoposide) with interim treatment with thalidomide and dexamethasone before high-dose melphalan and PBSC transplantation. All patients on Total Therapy III received thalidomide. Stem cell collection occurred twice, after the first and second cycles of chemotherapy, for two high-dose melphalan treatments. Each melphalan treatment (one after the second cycle of VDTPACE and another about two to three months later) was followed by PBSC transplantation.

**Table 2:** Data extraction sheet of included studies: article characteristics, intervention, and limitations.

Article Characteristics	Exercise Intervention Group				Usual Care/ Comparison Group	Study Completion	Limitations
	Supervision	Frequency and Duration	Modality	Adherence			
Mixed Populations Including Individuals with MM							
Andres et al. USA 2020 [30]	Unsupervised	Daily from admission to 60 days post-transplant	Aerobic + Resistance	Unknown	N/A	167 patients completed study; 40 patients died during data collection	Unsupervised exercise: adherence unknown. Each participant received the same exercise program.
Dimeo et al. Germany 2003 [32]	Supervised	Daily during hospitalization	Aerobic	Unknown	N/A	All patients completed the study, other than two who died due to sepsis	Results are generalized as only 12% of the participants had MM. No control group. Submaximal testing used and predictive equations used for estimating maximal oxygen consumption.
Furzer et al. Australia 2016 [36]	Partially supervised	3x/week, 12 weeks	Aerobic + Resistance	91% adherence to exercise (self-reported diaries)	UC group received general healthy lifestyle advice	Exercise group: 18/22, usual care: 19/22; total: 37/44 (84.1%)	Limited supervision. Adherence self-reported.
Hacker et al. USA 2016 [38]	Partially supervised	3x/week, 6 weeks	Resistance	83% adherence to exercise	UC group received recommendations regarding rest, physical activity, and exercise from their attending HCT physician	89% completed study of subjects initially enrolled	Short-duration study (6 weeks).
Persoon et al. The Netherlands 2017 [40]	Supervised	18 weeks total; 2x/week for first 12 weeks, 1x/week from week 13 onward	Aerobic and Resistance	Average attendance 25.8/30 (86%), with 75% attending greater than 80% of the exercise sessions	UC group was not restricted in their physical activities but was not specifically motivated to exercise	Intervention lost 4 (7%) to follow-up due to the progression of MM (2) or relapse (N)HL (2); usual care lost 8 (15%) to follow-up	Missing values for VO_2_peak (36%); contamination of control group exercising.
Rosko et al. USA 2021 [35]	Partially supervised	4 months; varied schedule	Aerobic, Resistance, and Balance	N/A	N/A	66.7% completion	No control group. High dropout rate. Details of exercise program not specified.
Tanaka et al. Japan 2017 [33]	Supervised	5x/week, 20 min/day	Aerobic and Resistance	N/A	N/A	N/A	Only one participant had MM.
**Studies Exclusive to Individuals with MM**							
Coleman et al., USA 2003 [31]	Unsupervised	Daily, 3 months pre- to 3 months post-SCT	Aerobic and Resistance	Exercise group completed exercise 75% of the time	UC group were to remain active and walk 20 min 3x/week	42% attrition; 1 death and 10 participants dropped out	Patients not supervised during exercise. High attrition rate.
Coleman et al. USA 2012 [34]	Unsupervised	Daily	Aerobic and Resistance	Unknown	UC group were asked to meet the current best practice (walking 20 min 3x/week)	N/A	Patients self-reported their adherence. Resistance prescription is ambiguous. Exercise specifics not clear.
Groeneveldt et al. United Kingdom 2013 [37]	Partially supervised	3x/week, 6 months	Aerobic, Resistance, and Mobility	First 3 months: logbook adherence of 86% ± 15%. Second 3 months: logbook adherence of 73% ± 24%.	N/A	49 entered the study; 37 completed the first 3 months (75.5%); 28 completed the full 6 months (57.1%)	Reduced logbook return rate, resulting in high probability of lower adherence than what was reported. No control group.
Koutoukidis et al. United Kingdom 2020 [39]	Partially supervised	3x/week, 6 months	Aerobic and Resistance	75% adherence to exercise; 80% of participants attended at least 50% of 12 classes in first 3 months.	UC group were asked to maintain their usual lifestyles	At 6 months, 76% remained in program	Exercise program specifics are not presented.
Purdy et al. Canada 2022 [41]	Partially supervised	2x/week RT, 90 min+/week aerobic, 12 weeks	Aerobic and Resistance	89.9% (group class) 82.9% (independent) 89.7% (aerobic)	N/A	26/28 completed the program (92.9%)	No control group. Gold standard measures not used due to virtual delivery. Required technology access and proficiency.

AMRAP: as many reps as possible.

**Table 3:** Data extraction sheet of included studies: criteria for sarcopenia in articles included.

Article Characteristics	Meeting Testing Criteria of EWGSOP2			Study Findings
	Muscular Strength	Muscle Quality	Muscle Performance	
Mixed Populations Including Individuals with MM				
Andres at al. USA 2020 [30]	X	X	✔ Timed up and go	Statistically significant differences for timed up and go test (*p* = 0.0031). Adverse events: Not reported.
Dimeo et al. Germany 2003 [32]	X	X	P Submaximal treadmill test	No significant differences in parameters related to the treadmill test after the intervention. Adverse events: Not reported.
Furzer at al. Australia 2016 [36]	✔ 5-RM for Leg, Chest, Back, and Arm Strength Sit-to-Stand	✔ DEXA	P Aerobic Power Index (API) Timed up and go Stair climb	No significant differences in timed up and go, sit-to-stand, or stair climb. Statistically significant differences in muscle strength measures at the 12-week and 24-week (all measures *p* ≤ 0.05) and body composition at 12 weeks (lean body and body fat% both *p* < 0.010) and 24 weeks (*p* ≤ 0.001 and *p* < 0.014). Adverse events: No adverse events occurred.
Hacker et al. USA 2016 [38]	✔ Hand Grip Arm Curl 30 s Chair Sit-to-Stand	✔ Thigh ultrasound	✔ Timed stair climb Timed up and go 15-foot walk time	No significant differences between groups in muscular strength (hand grip, arm curl, or chair sit-to-stand) or muscle quality as measured through ultrasound. Adverse events: Not reported.
Persoon et al. The Netherlands 2017 [40]	✔ Hand Grip 30 s Chair Sit-to-Stand	P Sum of four skinfolds	P VO_2_peak test on cycle ergometer	No significant between-group differences in any outcomes. Adverse events: Not reported.
Rosko et al. USA 2021 [35]	✔ Grip Strength 30 s Chair Sit-to-Stand	X	✔ Gait speed SPPB	SPPB median results improved to normal function at visit 2 (*p* < 0.001) and at visit 3 (*p* = 0.003). Adverse events: Not reported.
Tanaka et al. Japan 2017 [33]	✔ Grip Strength, Knee Extensor Strength	✔ Circumferences	P 6MWT	Statistically significant decreases in both handgrip (left *p* = 0.001 and right *p* = 0.006) and knee extensor strength (left *p* <0.001 and right *p* = 0.001). Both upper body and lower body circumference measurements were statistically significant (*p* < 0.05). No data reported for the 6MWT. Adverse events: Not reported.
**Studies Exclusive to Individuals with MM**				
Coleman et al., USA 2003 [31]	✔ 1-RM Leg Extension, Chest Press, Leg Press, Arm Pull, and Leg Curl	✔ Bod Pod	P Modified Balke	Statistically significant differences for lean body weight (*p* < 0.01). Adverse events: Not reported.
Coleman et al. USA 2012 [34]	X	X	P 6MWT	No significant difference in 6MWT distance. Adverse events: Serious adverse effects were similar for the control and HBIEP groups.
Groeneveldt et al. United Kingdom 2013 [37]	✔ Hand Grip 10 rep max knee extensor using leg press	✔ Bioelectrical Impedance	P 8 min submaximal single-stage walking test estimating VO_2_max	Upper and lower body limb strength improved significantly (both *p* < 0.001). Adverse events: No adverse events occurred.
Koutoukidis et al. United Kingdom 2020 [39]	✔ 10-RM Leg Extensor Strength, Grip Strength	✔ Bioelectrical impedance	P VO_2peak_ test on cycle ergometer	Statistically significant improvement in leg muscle strength (*p* = 0.03); VO_2_ peak significant in per-protocol analysis only. Adverse events: No serious adverse events; five participants with minor musculoskeletal events that were spontaneously resolved.
Purdy et al. Canada 2022 [41]	✔ 30 s Chair Sit-to-Stand	X	P 2-min step test	Statistically significant measures for both 2 min step test and 30 s chair sit-to-stand test (*p* < 0.05). Adverse events: No events met serious adverse criteria; three musculoskeletal events and one unrelated to exercise. No hospitalization for any events.

✔ = met criterion; X: did not meet criterion; *p* denotes physical tests that in the opinion of the authors, may be an acceptable alternative measure to satisfy the EWGSOP2 category criteria; RM: repetition maximum; 6MWT: six-minute walk test. SPPB: short physical performance battery.

## Data Availability

The original contributions presented in this study are included in the article/Supplementary Material. Further inquiries can be directed to the corresponding author.

## Supplementary Materials

The following supporting information can be downloaded at https://www.mdpi.com/article/10.3390/curroncol32100581/s1, S1: Search strategy.

## Abbreviations

The following abbreviations are used in this manuscript:

MM	Multiple myeloma
SCT	Stem cell transplant
EWGSOP2	European Working Group on Sarcopenia in Older People
CT	Computed tomography
MRI	Magnetic resonance imaging
DXA	Dual X-ray absorptiometry
SPPB	Short physical performance battery
TUG	Timed up and go test
VO_2peak_	Peak oxygen consumption
FITT	Frequency, intensity, type, and time
