# Supplementary material for: Exercise Interventions to Address Sarcopenia in People with Multiple Myeloma: A Scoping Review
Source: Curr Oncol. 2025 Oct 18;32(10):581. doi: 10.3390/curroncol32100581 (PMC12563198; doi:10.3390/curroncol32100581)
Supplement: Supplementary file 1 [file curroncol-32-00581-s001.zip › curroncol-3849801-supplementary.pdf]

Medline
Date searched: May 26, 2022, May 4, 2023, March 20/25
Results: 46

1. neoplasms, plasma cell/ or multiple myeloma/ or leukemia, plasma cell/
2. (Myeloma* or kahler* disease or plasma cell neoplasm* or plasma cell leukemia or plasma cell cancer or blood cancer or hematological cancer or haematogical cancer or bone marrow cancer or Paraproteinemia or malignant plasmacytoma or myeloproliferative disorder or hematologic malignanc* or haematologic malignanc*).mp.
3. 1 or 2
4. muscle weakness/ or muscular atrophy/ or sarcopenia/
5. Cachexia/
6. (Sarcopenia or cachexia or frail* or (muscle adj3 (weakness or atroph* or wasting))).mp.
7. 4 or 5 or 6
8. exercise/ or muscle stretching exercises/ or exp physical conditioning, human/ or running/ or jogging/ or swimming/ or walking/ or weight lifting/ or stair climbing/ or physical endurance/ or physical exertion/ or physical fitness/ or cardiorespiratory fitness/ or Dance Therapy/ or tai ji/ or yoga/ or exp exercise therapy/ or exercise movement techniques/
9. (exercis* or physical* activ* or physical* exert* or physical endurance or fitness or aerobic* or workout* or kinesiotherap* or stretches or stretching or core stability).mp.
10. ((conditioning or strengthening) adj6 (program* or protocol* or regime* or strateg* or therap* or intervention* or progressive or functional or home or home-based or outpatient)).mp.
11. ((train* adj6 (postur* or resist* or strength* or weight or stability or circuit* or interval* or endurance)) or (musc* adj6 (strengthen* or train* or contraction*)) or resistance activit* or progressive resist* or gravity resistive or stabilization exercis* or stabilisation exercis* or isotonic or isometric or ((eccentric or concentric) adj2 (contraction* or exercise*))).mp.
12. (activat* adj4 muscle).mp.
13. ((treadmill* not treadmill test) or (cardio* adj3 (class* or exercis* or train* or machine*)) or ergometer* or ergometre*).mp.
14. (walking or ((walk or walks) adj3 (fast or brisk* or quickly or regular)) or swim* or running or jogging or cycling or bicycl* or bike or biking or spinning or spin class* or spin bike* or x-country ski* or cross-country-ski* or ((stair* adj3 climb*) not stair climb test)).mp.
15. (danc* or calisthenic* or zumba or yoga or pilates or tai chi or tai ji).mp.
16. (pedomet* or acceleromet* or Step-count* or count*-step* or Fitness-tracker*).mp.
17. 8 or 9 or 10 or 11 or 12 or 13 or 14 or 15 or 16
18. 3 and 7 and 17

Embase
Date searched: May 26, 2022, May 4, 2023, March 20/25
Results: 264

1. malignant plasmacytoma/ or hematologic malignancy/
2. myeloma/ or myeloproliferative disorder/
3. paraproteinemia/ or smoldering multiple myeloma/

4. plasmacytoma/
5. plasma cell leukemia/
6. (Myeloma* or kahler* disease or plasma cell neoplasm* or plasma cell leukemia or plasma cell cancer or blood cancer or hematological cancer or haematogical cancer or bone marrow cancer or Paraproteinemia or malignant plasmacytoma or myeloproliferative disorder or hematologic malignanc* or haematologic malignanc*).mp.
7. 1 or 2 or 3 or 4 or 5 or 6
8. muscle weakness/
9. muscle atrophy/ or sarcopenia/
10. cachexia/
11. (Sarcopenia or cachexia or frail* or (muscle adj3 (weakness or atroph* or wasting))).mp.
12. 8 or 9 or 10 or 11
13. exp exercise/ or exp kinesiotherapy/ or dance therapy/ or physical activity/ or "physical activity, capacity and performance"/ or climbing/ or cycling/ or jogging/ or lifting effort/ or running/ or swimming/ or walking/ or weight bearing/ or weight lifting/
14. (exercis* or physical* activ* or physical* exert* or physical endurance or fitness or aerobic* or workout* or kinesiotherap* or stretches or stretching or core stability).mp.
15. ((conditioning or strengthening) adj6 (program* or protocol* or regime* or strateg* or therap* or intervention* or progressive or functional or home or home-based or outpatient)).mp.
16. ((train* adj6 (postur* or resist* or strength* or weight or stability or circuit* or interval* or endurance)) or (musc* adj6 (strengthen* or train* or contraction*)) or resistance activit* or progressive resist* or gravity resistive or stabilization exercis* or stabilisation exercis* or isotonic or isometric or ((eccentric or concentric) adj2 (contraction* or exercise*))).mp.
17. (activat* adj4 muscle).mp.
18. ((treadmill* not treadmill test) or (cardio* adj3 (class* or exercis* or train* or machine*)) or ergometer* or ergometre*).mp.
19. (walking or ((walk or walks) adj3 (fast or brisk* or quickly or regular)) or swim* or running or jogging or cycling or bicycl* or bike or biking or spinning or spin class* or spin bike* or x-country ski* or cross-country-ski* or ((stair* adj3 climb*) not stair climb test)).mp.
20. (danc* or calisthenic* or zumba or yoga or pilates or tai chi or tai ji).mp.
21. (pedomet* or acceleromet* or Step-count* or count*-step* or Fitness-tracker*).mp.
22. 13 or 14 or 15 or 16 or 17 or 18 or 19 or 20 or 21
23. 7 and 12 and 22

CINAHL Plus with Full Text

Date searched: May 26, 2022, May 4, 2023, March 20/25.

Results: 67

S1 (MH "Plasmacytoma+")

S2 (Myeloma* or kahler* disease or plasma cell neoplasm* or plasma cell leukemia or plasma cell cancer or blood cancer or hematological cancer or haematogical cancer or bone marrow cancer or Paraproteinemia or malignant plasmacytoma or myeloproliferative disorder or hematologic malignanc* or haematologic malignanc*)

S3 (MH "Muscle Weakness+") OR (MH "Muscular Atrophy+") OR (MH "Sarcopenia")
S4 (MH "Cachexia")
S5 (Sarcopenia or cachexia or frail* or (muscle N3 (weakness or atroph* or wasting)))
S6 (S1 OR S2) AND (S3 OR S4 OR S5)
S7 (MH "Exercise+") OR (MH "Physical Fitness") OR (MH "Physical Activity") OR (MH "Physical Performance") OR (exercis* or physical*-activ* or physical*-exert* or physical-endurance or fitness or aerobic* or workout* or kinesiotherap* or stretches or stretching or core stability or resistance-activit* or progressive-resist* or gravity-resistive or stabilization exercis* or stabilisation exercis* or isotonic or isometric or ((eccentric or concentric) N2 (contraction* or exercise*)) or ((conditioning or strengthening) N6 (program* or protocol* or regime* or strateg* or therap* or intervention* or progressive or functional or post-operative or postoperative or home or home-based or outpatient)) or (train* N6 (postur* or resist* or strength* or weight or stability or circuit* or interval* or endurance)) or (musc* N6 (strengthen* or train* or contraction* or activat*)) or treadmill* or (cardio* N3 (class* or exercis* or train* or machine*)) or ergometer* or ergometre* or walking or ((walk or walks) N3 (fast or brisk* or quickly or regular)) or swim* or running or jogging or cycling or bicycl* or bike or biking or spinning or spin class* or spin bike* or kickbox* or boxing or x-country ski* or cross-country-ski* OR (stair* N3 climb*) or rowing or skating or hiking or danc* or gymnastic* or calisthenic* or zumba or yoga or pilates or tai chi or tai ji or pedomet* or acceleromet* or Step-count* or count*-step* or Fitness-tracker*)
S8 S6 AND S7

Scopus (Advanced search)

May 4, 2023, March 20/23, March 20/25.
TITLE-ABS-KEY(Myeloma* or kahler*-disease or plasma-cell-neoplasm* or plasma-cell-leukemia or plasma-cell-cancer or blood-cancer or hematological-cancer or haematogical-cancer or bone-marrow-cancer or Paraproteinemia or malignant-plasmacytoma or myeloproliferative-disorder or hematologic-malignanc* or haematologic-malignanc*) AND TITLE-ABS-KEY(Sarcopenia or cachexia or frail* or (muscle W/3 (weakness or atroph* or wasting))) AND TITLE-ABS-KEY(exercis* or physical*-activ* or physical*-exert* or physical-endurance or fitness or aerobic* or workout* or kinesiotherap* or stretches or stretching or core-stability or resistance-activit* or progressive-resist* or gravity-resistive or stabilization-exercis* or stabilisation-exercis* or isotonic or isometric or ((eccentric or concentric) W/2 (contraction* or exercise*)) or ((conditioning or strengthening) W/6 (program* or protocol* or regime* or strateg* or therap* or intervention* or progressive or functional or post-operative or postoperative or home or home-based or outpatient)) or (train* W/6 (postur* or resist* or strength* or weight or stability or circuit* or interval* or endurance)) or (musc* W/6 (strengthen* or train* or contraction* or activat*)) or treadmill* or (cardio* W/3 (class* or exercis* or train* or machine*)) or ergometer* or ergometre* or walking or ((walk or walks) W/3 (fast or brisk* or quickly or regular)) or swim* or running or jogging or cycling or bicycl* or bike or biking or spinning or spin-class* or spin-bike* or kickbox* or boxing or x-country-ski* or cross-country-ski* OR (stair* W/3 climb*) or rowing or skating or hiking or danc* or gymnastic* or calisthenic* or zumba or yoga or pilates or tai-chi or tai-ji or pedomet* or acceleromet* or Step-count* or count*-step* or Fitness-tracker*)
